# Deletion 9p23 to 9p11.1 as sole additional abnormality in a Philadelphia positive chronic myeloid leukemia in blast crisis: a rare event

**DOI:** 10.1186/s13039-015-0165-0

**Published:** 2015-08-04

**Authors:** Abdulsamad Wafa, Manar Asa’ad, Adnan Ikhtiar, Thomas Liehr, Walid Al-Achkar

**Affiliations:** Department of Molecular Biology and Biotechnology, Human Genetics Division, Atomic Energy Commission, P.O. Box 6091, Damascus, Syria; Department of Molecular Biology and Biotechnology, Mammalians Biology Division, Atomic Energy Commission, Damascus, Syria; Institute of Human Genetics, Jena University Hospital, Jena, Germany

**Keywords:** Chronic myeloid leukemia, Philadelphia chromosome, del(9)(p24p12), *CDKN2A* gene, Prognostic factors

## Abstract

**Background:**

Chronic myeloid leukemia (CML) is a myeloproliferative disorder characterized by the presence of a derivative chromosome 22 [der(22)] commonly called Philadelphia chromosome (Ph). The Ph chromosome is a product of the reciprocal translocation t(9;22)(q34.1;q11.2). Additional genetic changes occur in less than 10 % of CML cases at the time of diagnosis and other genetic changes are seen in 60–80 % of the cases in advanced disease. Even though deletions in chromosome 9 are not rare findings in advanced phase-CML, del(9)(p23p11.1) as sole additional abnormality detected by fluorescence in situ hybridization (FISH) technique, to our knowledge has not been described in the literature.

**Results:**

A complete cytogenetic and molecular cytogenetic analysis, molecular biology method (reverse transcription polymerase chain reaction (RT-PCR)), and immunophenotype confirmed to be a CML case in blast crisis (BC). It revealed del(9)(p23p11.1) as sole abnormality detected by FISH technique besides Ph chromosome, which leads to monoallely of tumor suppressor gene *CDKN2A* (cyclin-dependent kinase inhibitor 2A) before Imatinib mesylate (IM) treatment.

**Conclusions:**

The patient did not demonstrate a good response to IM treatment. The underlying mechanisms and prognostic implications of these cytogenetic abnormalities are discussed.

## Background

Chronic myeloid leukemia (CML) is a myeloproliferative disorder. It is mainly characterized by the presence of a derivative chromosome 22 [der(22)], the so-called Philadelphia chromosome (Ph), which is due to a reciprocal translocation t(9;22)(q34.1;q11.2). This rearrangement involves the breakpoint cluster region (*BCR*) gene 22q11.2 and the c-Abelson (*ABL1*) gene 9q34, resulting in the BCR-ABL1 fusion gene, encoding a constitutively active tyrosine kinase protein [1].

The Ph chromosome can be observed as a sole chromosomal anomaly during the early chronic phase (CP) of CML (in more than 90 % of the CML cases), additional genetic changes occur in less than 10 % of cases at the time of diagnosis and other genetic changes are seen in 60–80 % of the cases in advanced disease [1]. The identification of this abnormality is important for the diagnosis of the disease determined by the WHO Tumor Classification [2] and for treatment purposes. The first therapeutic choice, tyrosine kinas inhibitors, has shown great therapeutic efficacy [3]. Imatinib mesylate (IM = Glivec, formerly STI571) is a chemically designed drug able to block BCR/ABL1 tyrosine kinase activity and is successfully used in CML patients [4].

Here we presented a de novo untreated Ph-positive CML case in BC phase with del(9)(p23p11.1) as sole additional abnormality detected by FISH technique, which leads to monoallely of tumor suppressor gene *CDKN2A* (cyclin-dependent kinase inhibitor 2A). This patient did not demonstrate a good response to IM treatment.

## Case presentation

In October 2013 a 40-year-old male was diagnosed as suffering from CML. Physical examination revealed the indicative symptoms as hepatosplenomegaly, loss of weight, fever, anemia, and thrombocytopenia. Routine peripheral blood test showed elevated white blood cells (WBC) of 243.1 × 10^9^/l (41 % of cells were blasts), red blood cell (RBC) count was 3.62 × 10^6^/mm^3^, hemoglobin level was 6.8 g/dl and the platelet count 26 × 10^9^/l. Serum lactate dehydrogenase value (LDH) was 1,543 U/l (normal level <460 U/l). The patient was diagnosed as CML-BC according to WHO recommendations, in a high Sokal risk of 29.7 (0.8–1.2), high Hasford (Euro) risk of 2,856 (>1,480), low Eutos risk of 40 (<87) and Etous probability of no complete cytogenetic response (CCgR) at 18 months was 15 %. In fact, the patient started on IM treatment (400 mg/day) for overall 1 month; then he increased IM daily dose to 800 mg for 1 month but the treatment was not successful and the patient had a toothache, anemia, and thrombocytopenia. Later he was given IM 400 mg daily dose for 2 months. Under the latter therapy the previous reported relevant symptoms disappeared. Unfortunately the patient died 7 months after diagnosis from the disease under the treatment for unknown reason.

## Results

**Fig. 1:**
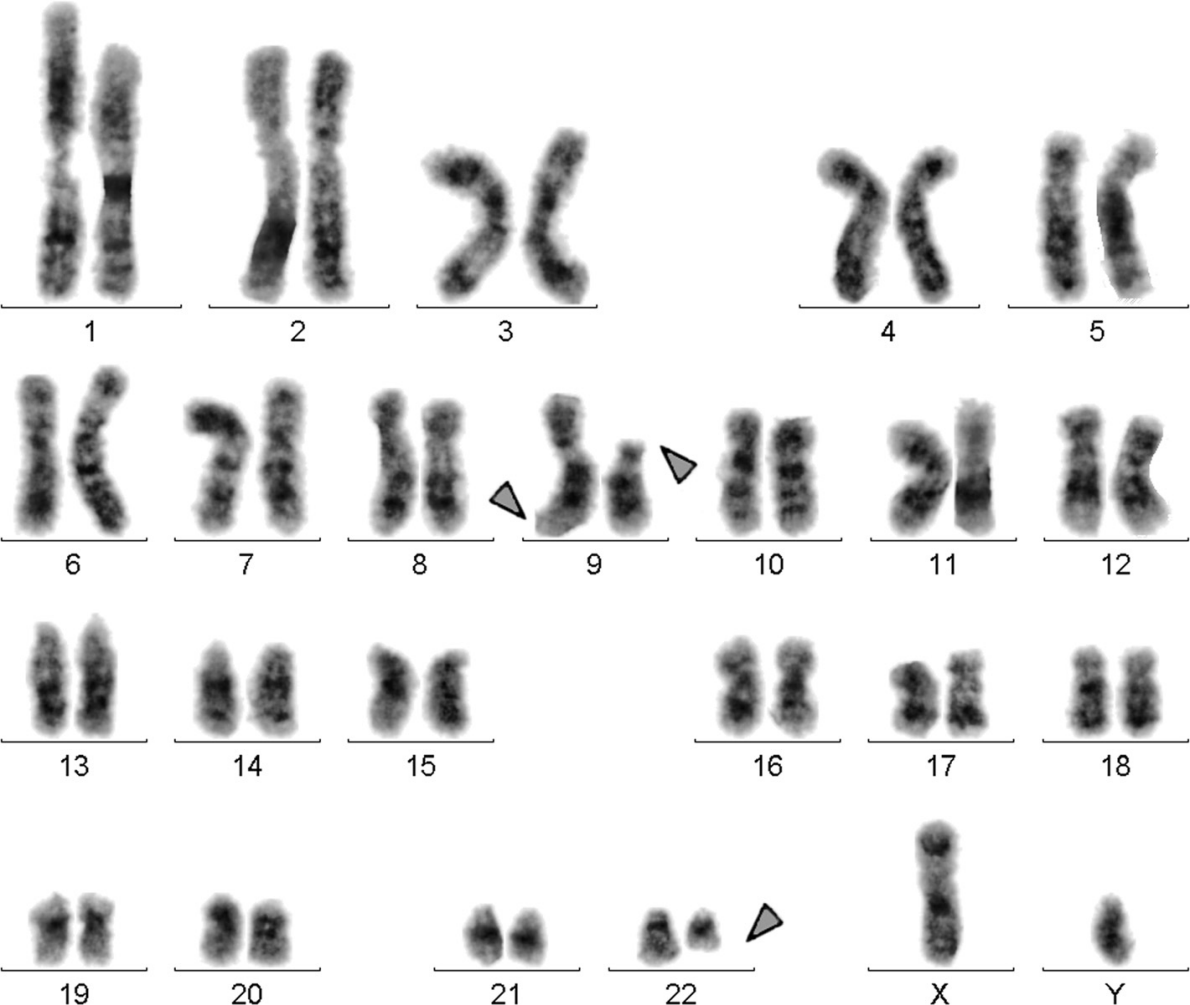
GTG-banding revealed a deletion of the short arm of a derivative chromosome 9 del(9)(p?). A derivative chromosome is marker by arrowhead

**Fig. 2:**
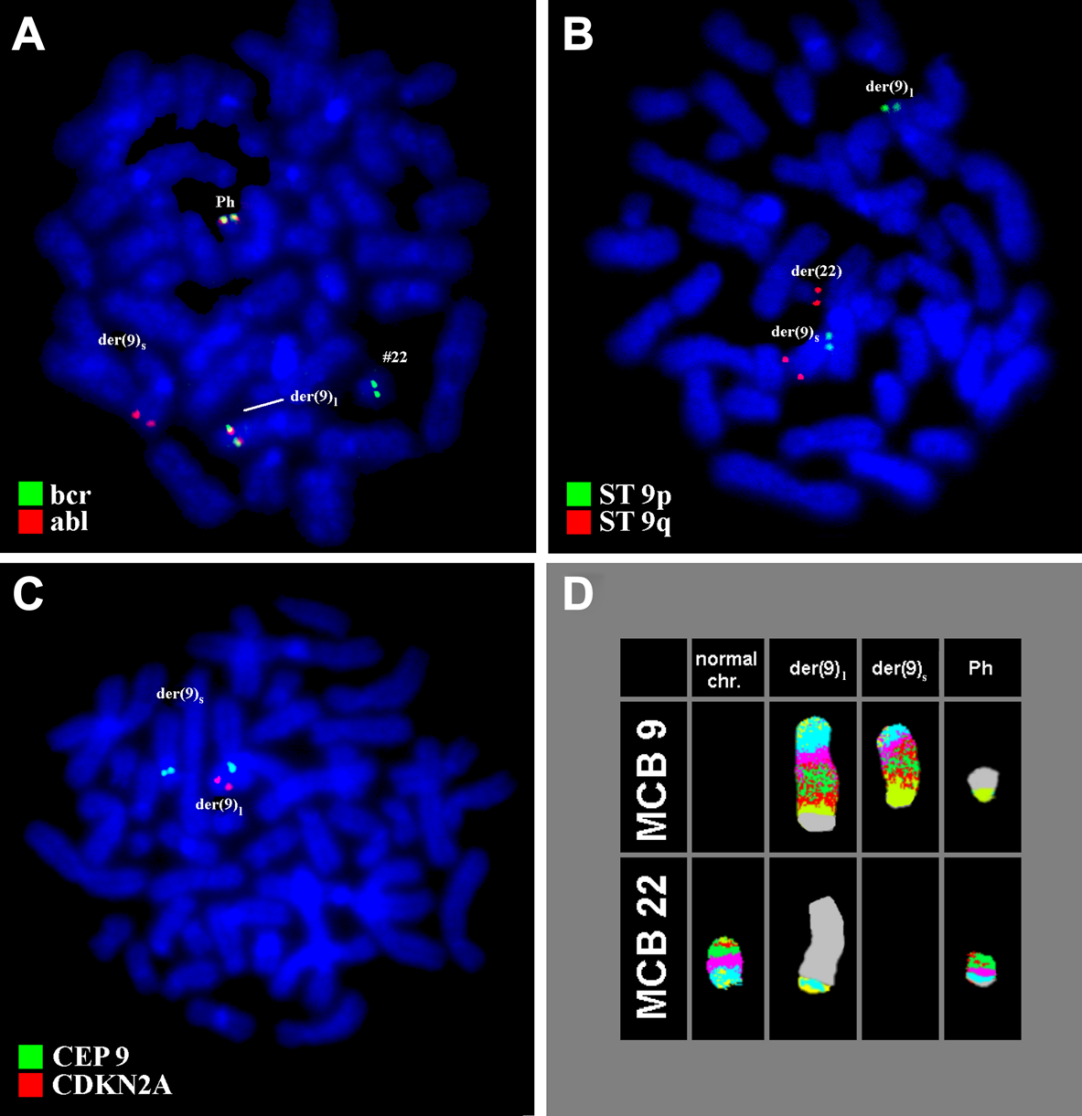
Karyotype and chromosomal aberrations were confirmed using molecular cytogenetic approaches. **a** FISH using probes for BCR (green) and ABL (red) showed 2 copies of BCR/ABL on Ph chromosome and on der(9), respectively. **b** FISH using probes for ST 9p (green) and ST 9q (red) showed; 1 green and 1 red signal on der(9); and 1 red signal on Ph chromosome and 1 green signal on der(9). **c** The deletion of *CDKN2A* was identified on the der(9). **d** The application of MCB 9 characterized the del(9)(p24p12) comprehensively. Abbreviations: # = chromosome; der = derivative chromosome

Prior to the IM-treatment GTG-banding revealed a karyotype of 46,XY,t(9;22)[11]/ 46,XY,t(9;22),del(9)(p?)[9] (Fig. 1). Further molecular cytogenetics studies were performed (Fig. 2). Dual-color-fluorescence in situ hybridization (FISH) prior to IM-treatment using a specific probe for BCR and ABL1 revealed two fusion signals, on the der(9) and der(22), respectively (Fig. 2a). A probe-set specific for subtelomeric (ST) regions in 9p and 9q revealed one green and one red signal on der(9), and one green, and one red signal on the der(9) and der(22), respectively (Fig. 2b). Chromosome 9 was studied with whole chromosome painting (WCP) probe and did not provide any hint on cryptic translocations (data not shown). A probe specific for *CDKN2A* confirmed that the deletion visible in the short arm encompassed subband 9p21 (Fig. 2c). Multicolor banding (MCB) using a specific probe for chromosome 9 confirmed a terminal deletion of 9p23 to 9p11.1 (Fig. 2d). RT-PCR pre IM treatment confirmed the presence of the BCR-ABL1 fusion (b2a2 transcript) revealing a major M-BCR transcript, most often identified in CML (data not shown). Thus, the following final karyotype prior IM-treatment was determined:

46,XY,t(9;22)(q34;q11.2)[11]/46,XY,der(9)(9pter- > 9p23::9p11.1- > 9q34::22q11.2- > 22qter),der(22)t(9;22)(q34;q11.2)[9].

The abnormal cell population (~42-45 % blasts in peripheral blood specimen) showed the following immunophenotype, which was consistent with CML-BC (WHO recommendations): CD45^+dim^, CD13^+^, CD33^+^, CD32^+^ ,CD22^+^, CD10^+^, CD41a^+^, CD34^−^, HLADr^−^, CD5^−^, CD7^-,^ CD2^−^, CD209^−^, CD11c^−^, CD79b^−^, sIgM^−^, sIgD^−^, CD103^−^, CD138^−^, CD56^−^, CD57^−^ and expressed CD64, CD38, and CD4 heterogeneously.

## Conclusions

We described a de novo untreated CML case in BC with del(9)(p23p11.1) as sole additional acquired abnormality, which leads to monoallely of tumor suppressor gene *CDKN2A* as located at 9p21. Up to date, there are 7 CML cases listed in Mitelman Database showed the involvement of the short arm of chromosome 9, two of them revealed involvement of 9p21 [5]. Also, loss of a part of the short arm of chromosome 9 (p12 and/or p21) in CML-BC are reviewed in two previous studies [5, 6]. To the best of our knowledge, this chromosomal abnormality del(9)(p23p11.1) has not been previously observed in CML [7].

According to the literature, approximately 10 % of CML patients present in advanced phases called accelerated phase (CML-AP) or CML-BC, without a clinically evident CP [8]. Even with the advent of tyrosine kinase inhibitor (TKI) therapy, the phase of disease remains an important prognostic factor in CML [9, 10].

The progression to advanced phases of disease is caused by the development of genetic lesions in addition to BCR-ABL1 translocation; secondary cytogenetic changes are identified in more than 80 % of patients by the time of progression to CML-BC [11]. Even in the absence of other features of CML-AP or CML-BC, the acquisition of new cytogenetic abnormalities in patients treated with IM is associated with loss of response and poor prognosis [12, 13].

The t(9;22) or a variant Ph are frequently the sole chromosomal anomaly during the CP [1]. The most common secondary chromosomal aberrations in advanced phase CML are +8, +Ph, i(17q), +19, −Y, +21, +17, −7, and −17 [1].

The median time to progression from CML-CP to CML-BC is 3–5 years in untreated patients or in patients treated with hydroxyurea [14]. However, BC is typically preceded by an intermediate AP of disease by a period of 4–6 months [15], but up to 25 % of CML-BC cases develop without any clinically evident preceding AP [16].

The molecular events associated with CML progression are complex and are likely related to genetic instability and impaired DNA repair mechanisms that are consequences of BCR-ABL1 activity [17–19]. In addition to cytogenetic aberrations, BCR-ABL1 amplification, gene mutations, and epigenetic changes such as gene methylation also appear to play a role in disease progression [20]. Interestingly, different BC phenotypes have been associated with unique patterns of molecular evolution: ABL kinase domain mutations that confer resistance to TKIs occur frequently in lymphoid BC, while myeloid BC is more commonly associated with cytogenetic evolution. BCR-ABL1 amplification is seen in about 31 % of both myeloid and lymphoid BC cases [21].

*CDKN2A* and *CDKN2B* are tumor suppressor genes located in 9p21. They belong to the family of inhibitors of cyclin-dependent kinases. The *CDKN2A* gene encodes two proteins, p14^ARF^ and p16^INK4a^, and the *CDKN2B* gene p15 ^INK4b^ protein, which as key regulators of G1 phase cell-cycle arrest and senescence [22]. These genes product is inactivated in a wide range of human cancers through epigenetic mechanisms [22, 23],

Deletion at 9p21 is especially frequent in acute lymphocytic leukemia (ALL), occurring at more than of 20 % in B-cell precursor ALL and approximately 50 % T-ALL patients [24]. The 9p21 deletions have been suggested to be associated with unfavorable outcome in both adult ALL and pediatric ALL, although the prognostic impact of *CDKN2A* deletions in pediatric ALL appears controversial [24–27].

Recently, FOXA1 is a key transcription (TF) factor for *CDKN2A* expression [23, 28]. FOXA1 is a member of forkhead family TFs with remarkable pioneering activity to open closed chromatin for its subsequent cooperation with other master TF in embryogenesis and organ development [29, 30].

In conclusion, we described de novo untreated CML case in BC with del(9)(p23p11.1) as sole additional acquired abnormality detected by FISH technique, which leads to monoallely of tumor suppressor gene *CDKN2A* as located at 9p21. The patient did not demonstrate a good response to Imatinib treatment.

## Materials and Methods

### Chromosome analysis

Chromosome analysis applying GTG-banding according to standard procedures [31] was performed prior IM treatment. 20 metaphase cells derived from unstimulated bone marrow culture were analyzed. Karyotypes were described according to the International System for Human Cytogenetic Nomenclature (ISCN 2009).

### Molecular cytogenetics

Fluorescence in situ hybridization (FISH) using the LSI BCR/ABL dual color dual fusion translocation probe (Abbott Molecular/Vysis, Des Plaines, IL, USA), a locus specific probe for CDKN2A gene (LSI p16 in 9p21) with a probe for centromere nine, and a subtelomeric probe for 9p and 9q (Abbott Molecular/Vysis, Abbott Park, IL, USA) were applied together with whole chromosome painting (WCP) probe for chromosome 9 (MetaSystems, Altlussheim, Germany) according to manufacturer’s instructions [31]. Also a multicolor banding probe (MCB) sets based on microdissection derived region-specific libraries for chromosome 9 was applied as previously described [32]. A minimum of ten metaphase spreads was analyzed, using a fluorescence microscope (AxioImager.Z1 mot, Carl Zeiss Ltd., Hertfordshir, UK) equipped with appropriate filter sets to discriminate between a maximum of five fluorochromes plus the counterstain DAPI (4',6- diamino-2-phenylindole). Image capture and processing were performed using an ISIS imaging system (MetaSystems, Altlussheim, Germany).

### Reverse transcriptase-polymerase chain reaction (RT-PCR) and for BCR/ABL1 fusion transcripts

Total RNA extracted from peripheral blood sample using the InviTrap RNA kit (Invitek, Berlin, Germany) according to the manufacturer’s recommendations. cDNA was prepared from 5 μg of total RNA with the Genequality BCR-ABL1 kit (AB Analitica, Padova, Italy) and BCR-ABL1 fusion transcript was performed according to the manufacturer’s instructions (AB Analitica, Padova, Italy).

### Flow cytometric immunophenotype

Flow cytometry of leukemic blasts was performed using a general panel of fluorescent antibodies against the following antigens typical for different cell lineages and cell types: CD1a, CD2, CD3, CD4, CD5, CD8, CD10, CD11b, CD11c, CD13, CD14, CD15, CD16, CD19, CD20, CD22, CD23, CD32, CD33, CD34, CD38, CD41a, CD45, CD56, CD57, CD64, CD103, CD117, CD123, CD209, CD235a and CD243; in addition antibodies against Kappa and Lambda light Chains, sIgD, sIgM, and HLADr were applied (BD Biosciences). Four-color immunophenotyping on peripheral blood specimen was performed. Samples were stained and analyzed on a BD FACSCalibur™ flow cytometer according to BD Biosciences manuals and products insert sheets. Autofluorescence, viability, and isotype controls were included. Flow cytometric data acquisition and analysis were conducted by BD Cellquest™ Pro software.

## Consent

Written informed consent was obtained from the patient for publication of this Case Report. A copy of the written consent is available for review by the Editor-in-Chief of this journal.
